# The Medical Formulary: Being a Collection of Prescriptions Derived from the Writings and Practice of Many of the Most Eminent Physicians in America and Europe

**Published:** 1846-11

**Authors:** 


ART. IV.—*The Medical Formulary:* being a Collection of Prescriptions derived from the Writings and Practice of many of the most eminent Physicians in America and Europe. To which is added an Appendix containing the usual Dietetic Preparations and Antidotes for Poisons. The whole accompanied with a few brief Pharmaceutic and Medical Observations. By Benjamin Ellis, M.D., late Professor of Materia Medica and Pharmacy in the Philadelphia College of Pharmacy. Eighth edition, with numerous additions. By SAMUEL GEORGE MORTON, M.D. Philadelphia: Lea & Blanchard. 8vo. pp. 272. 1846.

This very ample title-page expresses the character of the Formulary, and leaves nothing for us to say concerning its scope and aim. The fact that it has gone to its eighth edition is the most substantial of all kinds of evidence, that it has been found useful to the profession. The present edition is a decided improvement upon all the preceding ones, and the work may be consulted with full confidence that it brings its subjects up to the science of the day. Whatever has been added by chemistry or practical medicine to the stores of our knowledge will be found embraced within its pages. The young physician desirous of writing neat prescriptions for his patients will turn to the pages of the "Medical Formulary" with great satisfaction.

